# Recent Progress in Interferon Therapy for Myeloid Malignancies

**DOI:** 10.3389/fonc.2021.769628

**Published:** 2021-10-29

**Authors:** Fiona M. Healy, Lekh N. Dahal, Jack R.E. Jones, Yngvar Floisand, John F. Woolley

**Affiliations:** ^1^ Department of Pharmacology & Therapeutics, University of Liverpool, Liverpool, United Kingdom; ^2^ Department of Molecular & Clinical Cancer Medicine, University of Liverpool, Liverpool, United Kingdom; ^3^ The Clatterbridge Cancer Centre NHS Foundation Trust, Liverpool, United Kingdom

**Keywords:** interferon, myeloid malignancies, interferon alfa, interferon beta, type I interferon (IFN) signaling, myeloid neoplasia

## Abstract

Myeloid malignancies are a heterogeneous group of clonal haematopoietic disorders, caused by abnormalities in haematopoietic stem cells (HSCs) and myeloid progenitor cells that originate in the bone marrow niche. Each of these disorders are unique and present their own challenges with regards to treatment. Acute myeloid leukaemia (AML) is considered the most aggressive myeloid malignancy, only potentially curable with intensive cytotoxic chemotherapy with or without allogeneic haematopoietic stem cell transplantation. In comparison, patients diagnosed with chronic myeloid leukaemia (CML) and treated with tyrosine kinase inhibitors (TKIs) have a high rate of long-term survival. However, drug resistance and relapse are major issues in both these diseases. A growing body of evidence suggests that Interferons (IFNs) may be a useful therapy for myeloid malignancies, particularly in circumstances where patients are resistant to existing front-line therapies and have risk of relapse following haematopoietic stem cell transplant. IFNs are a major class of cytokines which are known to play an integral role in the non-specific immune response. IFN therapy has potential as a combination therapy in AML patients to reduce the impact of minimal residual disease on relapse. Alongside this, IFNs can potentially sensitize leukaemic cells to TKIs in resistant CML patients. There is evidence also that IFNs have a therapeutic role in myeloproliferative neoplasms (MPNs) such as polycythaemia vera (PV) and primary myelofibrosis (PMF), where they can restore polyclonality in patients. Novel formulations have improved the clinical effectiveness of IFNs. Low dose pegylated IFN formulations improve pharmacokinetics and improve patient tolerance to therapies, thereby minimizing the risk of haematological toxicities. Herein, we will discuss recent developments and the current understanding of the molecular and clinical implications of Type I IFNs for the treatment of myeloid malignancies.

## Clonal Malignancies in Myeloid Lineages

Myeloid malignancies, consisting of myeloproliferative neoplasms (MPNs), myelodysplastic syndrome (MDS), and acute myeloid leukaemia (AML), are a heterogeneous group of clonal haematopoietic disorders caused by abnormalities in HSCs and myeloid progenitor cells, originating in the bone marrow niche (1, 2). Each myeloid malignancy is unique and therefore presents different challenges with regards to treatment. MPNs have been categorized by the WHO as: chronic myeloid leukaemia (CML), polycythaemia vera (PV), primary myelofibrosis(PMF) and essential thrombocythemia (ET) and are characterized by the presence of the *BCR-ABL* gene, *JAK2*, *MPL* or *CALR* mutations, excessive erythrocyte production, fibrotic bone marrow degeneration, and excessive megakaryocyte lineage proliferation, respectively (3). MDS is characterized by abnormalities in normal haematopoiesis with resulting progression to bone marrow failure and genetic instability with potential to develop into AML (4) which is often considered the most aggressive myeloid malignancy. Older AML patients, those with poor-risk associated karyotypes, or with an unfavourable mutational burden (e.g. *TP53* mutations) have a median survival as low as 4-6 months (5–7). In comparison, patients who are diagnosed with CML and treated with tyrosine kinase inhibitors (TKIs), such as imatinib, have an overall long-term (8-year) survival rate as high as 93% (8–10). This vast difference in survival between different malignancies warrants a novel and innovative approach to the treatment of certain myeloid malignancies.

There are currently a variety of therapies available for myeloid malignancies, dependent on the disease in question. Most CML patients will experience long term responses with tyrosine kinase inhibitors (TKIs) targeting the *BCR-ABL* oncogene and can in many cases be considered functionally cured of their disease (9, 11). PV is associated with blood hyperviscosity due to the expansion of the erythrocyte mass, and therefore standard therapy involves blood withdrawal to reduce mass as well as treatment with hydroxyurea, or ruxolitinib (Janus activated kinase 1/2 (JAK1/JAK2) inhibitor) as a second-line chemotherapy option (12, 13). PMF can also be treated using JAK2 inhibitors, however this is considered only a symptom relieving measure – PMF is only potentially curable with an allogeneic haematopoietic stem cell transplant (HCT) and is associated with a substantial risk of treatment-related mortality (14). Finally, MDS and AML can also be treated with high dose, aplasia-inducing chemotherapy and consolidated with HCT. However, relapse remains a problem and many factors such as poor cytogenetic risk (15); monosomal karyotype (16); measurable residual disease (MRD) positivity (17, 18); FLT3-ITD and other mutations (19–21) can influence the risk of relapse in AML patients after HCT.

A large portion of AML patients are elderly and are considered unfit to undergo intensive chemotherapy (22). Current treatment for these malignancies is therefore unfavourable for many patients. Alternatively, hypomethylating agents in combination with BCL2 inhibitors (23, 24) are becoming an increasingly better option to reduce toxicity (25), although a new treatment for MDS and AML is still urgently required.

For decades, cytokines belonging to the Interferon (IFN) family have been shown to play an integral role in co-ordinating immune responses against viruses, intracellular pathogens and tumour control (26). IFNs are divided into three types: Type I (including IFN-α and IFN-β), Type II (IFN-γ only) and Type III (IFN-λ). A summary of these subtypes and potential therapeutics is described in **Table 1** (27–29). Type I IFNs are produced by most cells, following detection of pathogen-associated molecular patterns (PAMPs), such as foreign or self-nucleic acids (30). Interestingly, Type I IFNs differ in their binding affinity to the same cell surface receptor (IFNAR1/IFNAR2) and consequently trigger different antiviral, antiproliferative, and immunomodulatory outcomes (31). It is these antiproliferative and immunomodulatory outcomes which highlight IFNs as a potential treatment for myeloid malignancies (32). Herein we discuss to what extent IFNs can be used therapeutically to manage myeloid malignancies.

**Table 1:** Summary of the Canonical Functions of Type I Interferons and Therapeutic Potential.

Type	Expressed in humans?	Function/Key Processes	Therapeutically used?	Cancer therapeutic?
IFN-α	Yes	Apoptosis, CD8+ T cell priming, antigen presentation, pro-inflammatory cytokine production	Yes- IFN-α2a, IFN- α2b IFN- α14	IFN- α2a, IFN- α2b
IFN-β	Yes	Anti-proliferative, anti-angiogenic, stimulation of immune response	Yes	Yes
IFN-δ	No	Anti-proliferative effects	No	No
IFN-ϵ	Yes	TNF- α pathway activation, ROS generation	No	No
IFN-ζ (Limitin)	No	MHC class I expression, increases cytotoxic T lymphocyte activity, anti-proliferative effects	No	No
IFN-κ	Yes	Increases IFN-β production	No	No
IFN-τ	No	IL-6/IL-8 expression and secretion	No	No
IFN-ω	Yes	MHC I molecule production, innate immune cytokine production, phagocytosis	Yes - veterinary uses	No

## Type I Interferon Signaling

**Figure 1:**
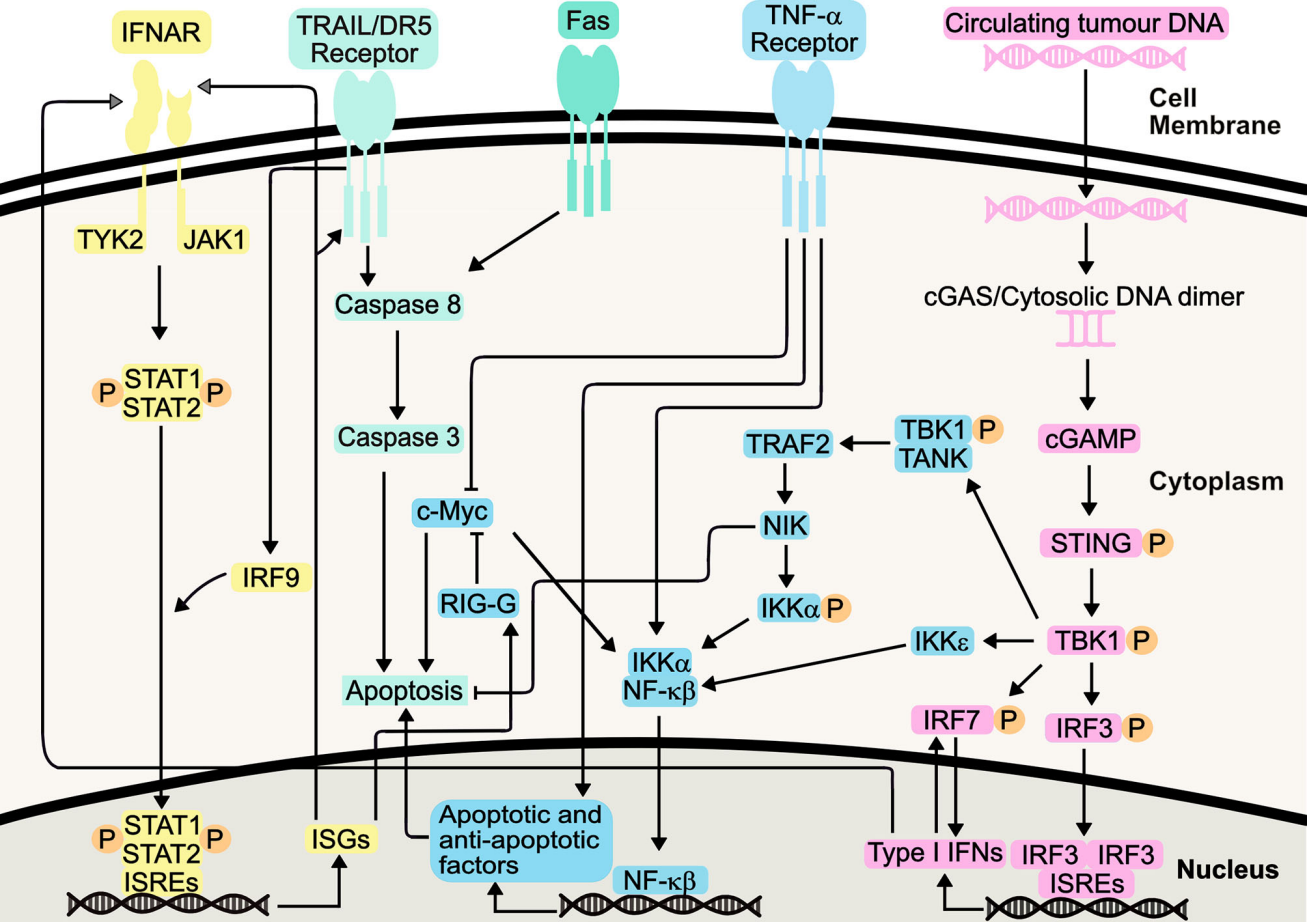
Type I Interferon Signalling Pathways. Type I IFNs bind the IFNAR1/IFNAR2 receptors, causing heterodimerization and TYK2 and JAK1 activation, initiating STAT1/STAT2 heterodimerization and phosphorylation. Following IRF-9-mediated translocation of this heterodimer to the nucleus, it binds interferon-stimulated response elements (ISREs), resulting in transcription of interferon-stimulated genes (ISGs). Resulting proteins include death receptor ligands (including TRAIL and Fas), which bind their respective receptors. Apoptosis is induced, either directly, or indirectly *via* NF-κβ signalling. These pathways also synergize with TNF-α pathway, increasing apoptosis. The STING pathway, initiated by detection of circulating tumour DNA, can mediate production of Type I IFNs or induce apoptosis, through canonical and non-canonical pathways following TBK1 activation. This includes its role in positive feedback loops involving IRF7, further increasing Type I IFN production.

Following binding of Type I IFNs to the IFNAR1/IFNAR2 receptor complex, the associated JAK1 and tyrosine kinase 2 (TYK2) activation causes the tyrosine phosphorylation of STAT1 (signal transducer and activator of transcription 1) and STAT2 (**Figure 1**). This ultimately results in the formation of an IFN-stimulated gene factor 3 (ISGF3) complex which is translocated to the nucleus. Here, it binds IFN-stimulated response elements (ISREs), initiating transcription of IFN-stimulated genes (ISGs) (33). These ISGs have a wide range of functions, and can be involved in immune system regulation and cell death, amongst other processes. ISGs are also involved in both positive and negative feedback loops within the IFN signalling system, with the potential to increase or decrease IFN signalling, depending on cellular context such as mutational status (34).

IFN signaling results in the transcription and translation of cell death-inducing ISGs, and it has been hypothesized that IFN-α works by potentiating Tumour Necrosis Factor (TNF)-induced apoptosis through suppression of nuclear factor-κβ (NF-κβ) (35). Co-treatment of AML cell lines with TNF-α and IFN-α increased apoptosis compared to IFN-α alone, indicating IFN-α enhancement of TNF-α apoptosis *via* distinct and common pathways (36). Increased IFN production has been shown to inhibit activity of NF-κβ through the NIK/TRAF2 pathway, yet, this can be somewhat of a ‘double-edged sword’, with this pathway also implicated in cellular protection against IFN-mediated apoptosis (37). Furthermore, IFN-mediated NF-κβ inhibition leads to downregulation of cellular Myc (c-Myc) by increasing *IFIT3* (*RIG-G*) gene expression, promoting apoptosis and inducing cell cycle arrest, *via* enhancement of CD95/Fas expression, triggering increased cellular caspase 3 activity (38–40). Indeed, IFN-α has been known to sensitize cells to TRAIL-induced apoptosis, through activation of DR5 receptor-mediated death signaling and subsequent suppression of TRAIL-mediated NF-κβ activation (41).

NF-κβ is also responsible for the production of cytokines in response to the detection of cytosolic DNA. Through the stimulator of interferon genes (STING) pathway, activation of interferon regulatory factor 3 (IRF-3) and NF-κβ leads to transcription of ISGs and cytokines. This includes IFN-α and IFN-β, and STING activation ultimately promotes varying mechanisms of cell death, including apoptosis (42–44). It can therefore be argued that the STING pathway activation of NF-κβ forms an opposing mechanism, perhaps to maintain homeostasis as well as desensitizing the body to further IFN stimulation (45). This could also explain why some IFN therapies require frequent administration of IFNs to maintain a therapeutic effect. Nevertheless, the STING pathway presents itself as a promising target to promote immunity in cancer, including the treatment of AML (46).

It has been proposed that antiproliferative effects stimulated by Type I IFN on cancerous cells can enhance the immune response in mouse models of haematological malignancies (47), through induction of cell cycle arrest. This is largely through an increase in p21 activity, as well as CDK2 inhibition, though perhaps to a lesser extent (48, 49). Type I IFN-mediated cell cycle arrest in S phase appears to be regulated through IRF9 (48–50). Increased levels of IRF9 have been attributed to increased *TRAIL* activity, which itself is an ISG (50, 51).

Type I IFNs can also negatively regulate angiogenesis, thereby reducing oxygen supply to malignant cells. This can be through increased transcription of anti-angiogenic factors, including the ISGs *IRF7* and *PML.* Alternatively, Type I IFNs can reduce production of pro-angiogenic factors, including basic fibroblast growth factor (bFGF), a factor increased in myeloid leukaemia (52, 53). Furthermore, it has been suggested that IFN therapy could reduce levels of pro-angiogenic vascular epidermal growth factor (VEGF), which is a biomarker used for CML severity (54, 55). Again, this primarily occurs through IFN-mediated reduction in *VEGFA* and *HIF1α* transcription, with lower levels of *VEGF* mRNA detected in tumour samples following Type I IFN therapy, as compared to diagnostic samples (52, 56).

Immunomodulatory effects of Type I IFNs can be mediated through dendritic cells, with Type I IFNs driving activation and maturation of dendritic cells (57). Following IFN-stimulated dendritic cell maturation, there can be an increase in MHC molecule production and antigen presentation to CD8+ T cells, thereby promoting CD8+ T cell mediated responses, ultimately leading to tumour destruction (58). Furthermore, IFN-stimulated dendritic cell maturation also induces expression of costimulatory ligands CD80 and CD86 (59–61). CD80 and CD86 are needed for full T cell activation, through their binding to CD28. Furthermore, increased CD80 expression induces IRF-7 expression, thereby stimulating further Type I IFN production, in somewhat of a positive feedback mechanism. Such pathway can be mediated by TRADD, TRAF3 and TANK activation, as well as IFN-β and IRF3 (31, 60, 62). Aside from these aforementioned signalling pathways, IFN-α also increases production of pro-inflammatory cytokines, including IL-1β, IFN-γ and TNF-α (31, 63). As previously stated, increased TNF-α contributes to increased apoptosis seen in response to Type I IFN. Thus, it can be deduced that, unsurprisingly, there is synergy between the various IFN-stimulated pathways, converging in key elements responsible for the apoptotic, anti-angiogenic, immunomodulatory and antiproliferative responses.

## Do IFNs Reduce the Rates of AML Relapse?

Treatment options for AML patients rely heavily on a combination of anthracyclines (e.g. daunorubicin, idarubicin) and the pyrimidine nucleoside cytarabine (64), which deliver an unsatisfactory 5-year survival rate of <20% in older patients (65–68). A goal of treating AML is obtaining a complete remission and preferably eradicating MRD, which is defined as the presence of residual leukaemic cells in bone marrow (BM) or peripheral blood (PB) in patients who have achieved morphologic complete remission (69). MRD positivity is associated with increased relapse risk and shorter survival in AML (70–73). One recent study found that measuring the transcript levels of *RUNX1/RUNX1T1* fusion gene in AML patients with MRD (post-HCT) allowed for a significant discrimination between patients that would continue remission or enter relapse (74). Other studies have supported this finding and argue that monitoring MRD using transcript levels of *RUNX1/RUNX1T1* fusion gene is favourable over the measurement of other mutations including *c-KIT* mutations for the risk stratification of AML patients with MRD (75). IFN therapy could potentially target the leukaemic stem cells (LSCs) at the root of AML development and relapse. It has been demonstrated that IFN-α triggers cell cycle entry in dormant HSCs (76). This raises the possibility that treating AML patients with IFN could force quiescent LSCs to proliferate, and thus amenable to standard chemotherapy. It has been shown that MRD-directed IFN-α treatment was able to significantly decrease the risk of cumulative incidence of relapse and improve survival in MRD-positive AML patients post-HCT, compared to MRD patients with no intervention (77, 78). Indeed, a recent retrospective study examined the use of IFN maintenance therapy following induction and consolidation chemotherapy in favourable-risk AML patients. Of patients who were MRD positive at baseline measurements, 78% of those treated with IFN-α2b became MRD negative, after a median time of 5.5 months. In contrast, only 27% of patients not treated with IFN-α2b (monitored only) showed MRD conversion from positive to negative. This corresponded to an increase in relapse-free survival, with 87% IFN-α2b treated patients relapse free after 4 years, compared to 56% in the control group (79). An ongoing phase I/II trial has demonstrated that pegylated IFN-α, when administered prophylactically after myeloablative conditioning in an AML cohort at high risk for relapse, did not significantly alter toxicity or acute graft *versus* host disease (GVHD) risk and produced relatively low rates of relapse suggesting a robust graft *versus* leukaemia (GVL) response (80). Separately a recent trial has explored the use of IFN-α2b therapy following MRD detection post induction and consolidation chemotherapy. A significant decrease in the number of patients with MRD in the IFN-α2b treated group was reported after a median follow-up time of 5 months, compared to a significant increase in patients with MRD detection in the trial arm that did not receive IFN after chemotherapy (patients observed only) (81). Importantly, event-free survival was also considerably higher in the IFN-α2b treated patients. IFN therapies to prevent relapse in AML potentially offer significant benefit to AML patients and this warrants further study.

## IFNs for TKI Resistant CML Patients

TKIs including imatinib, nilotinib, dasatinib, bosutinib, and most recently ponatinib, are the current preferred treatment for CML patients, and have dramatically improved the outcome of CML patients (82). Despite the success of TKIs, there is still a possibility that some CML patients become resistant to front-line treatment with imatinib, and to second generation TKIs (e.g. nilotinib, dasatinib) through the *BCR-ABL* T315I mutation wherein only third generation TKIs (e.g. ponatinib) are efficacious. Even still, resistance to imatinib has been seen since the earliest trials and has recently been shown for third-generation TKIs (83, 84). IFN therapy for the treatment of CML pre-dates TKI therapy, and is characterized by its ability to induce haematologic remission and durable cytogenetic response (85). Combination therapy of IFNs with cytarabine for many CML patients showed promising results, however the advent of TKIs meant that IFN therapy was largely replaced (86). The IRIS trial compared the use of imatinib or IFN-α plus cytarabine. Whilst there was no significant difference in survival after 18 months between either group, patients treated with IFN-α and cytarabine showed a markedly poorer cytogenetic response, coupled with a greater risk of disease progression, compared to those who received imatinib alone. Indeed, many patients who initially received IFN-α and cytarabine crossed over to imatinib therapy (9, 87, 88). Response to imatinib continued over 5 years, with a complete cytogenetic response seen in 69% of all patients who received imatinib (and up to 96% in those still taking imatinib after 5 years) (87). However, approximately 17% patients did lose complete cytogenetic response over the follow-up period (9).

This is in line with current understanding which suggests that TKIs are not entirely curative as monotherapies in CML, as LSC exposure to many TKIs fails to induce apoptosis in the most primitive quiescent CML LSCs (89, 90). Combined therapy of IFN-α with TKIs could address this issue by stimulating cytotoxic T cells to target LSCs refractory to standard TKI therapy. Initial studies comparing patients treated with imatinib and IFNα, to those treated with imatinib alone, showed that the combination therapy resulted in a higher rate of complete cytogenetic remission at 6 months, and similar rates at 4 years (91). Similarly, a trial of imatinib and IFN-α combination therapy showed 75% of patients showed sustained remission following imatinib discontinuation at 2.4 years (92). However, other trials involving the combination of IFNs with imatinib have shown mixed results, with no improvement in cytogenetic response rates or progression-free survival and increased adverse events (93, 94). For example, in extended (10 year) follow-up of the aforementioned IRIS trial, patients treated with imatinib alone had similar overall and progression-free survival than those treated with imatinib and IFN-α (95).

Whilst imatinib is considered a ‘game-changing’ drug for the extremely high survival rate it is associated with, second generation TKIs have shown further improvements in these rates in certain cases (10). IFN-α combination therapy with the second generation TKI dasatinib has also shown promising results (96). Low dose pegylated IFN-α following dasatinib treatment was well tolerated, and demonstrated rapid increased response rates with major molecular response (MMR) rate of 89% after 18 months. Encouragingly pegylated IFN-α was well tolerated in this study, and further investigation is warranted with second generation TKIs. Earlier studies of combination therapy comprising pegylated IFN-α formulations with imatinib, had shown good clinical response (significantly higher MMR of 82% compared to 54% for imatinib monotherapy after 12 months), yet were associated with toxicity also in a majority of patients (54). NiloPeg, a small 2011 phase II trial looking at the use of nilotinib and IFN-α2a showed a good molecular response in patients treated with this regimen (64% after 12 months), although adverse effects were, again, a frequent occurrence (97). The currently ongoing TIGER trial of nilotinib combination therapy with pegylated IFN-α is attempting to address these concerns, and will evaluate the feasibility of discontinuing drug therapy in patients showing stable deep molecular response after nilotinib *versus* IFN maintenance therapy (98).

## Treating MPNs With IFNs

The WHO classification system for MPNs includes seven subcategories (3), but more commonly this refers to the *JAK2* mutation-enriched diseases polycythaemia vera (PV), essential thrombocythaemia (ET) and primary myelofibrosis (PMF). PV is characterized by an elevated red blood cell mass (and potentially overproduction of white blood cells and platelets). PV is also associated with an increased risk for thromboembolic events, leukaemic transformation, and/or myelofibrosis.

Type I IFN signalling can contribute to the pathology of MPN, as well as being used as a therapeutic, as mentioned previously. In PMF, levels of IFN-α, and associated proinflammatory cytokines such as IL-1β, can be increased compared to healthy bone marrow or plasma. This contributes to the inflammatory phenotype associated with PMF (99). This coincides with the effects of *TET2* mutations, which are common in MPNs and are also associated with inflammation (100). In a similar way, whilst myelodysplastic syndrome (MDS) has a vast range of causes, increased pro-inflammatory signalling is thought to be a key driver, again mediated by Type I IFNs. Type I IFN-associated genes were found to be upregulated in MDS patients’ bone marrow, including *IRF-7* and *ISG-15*, suggesting their involvement in MDS pathology (101).

Standard therapy for PV involves phlebotomy and cytoreductive drugs (hydroxyurea, busulfan) but recently IFN-α and JAK1/2 inhibitors have been examined as potential treatments (102, 103). Although busulfan has been effective over long periods (104), resistance to hydroxyurea is associated with increased mortality and progression to more advanced MPNs or secondary AML (13). IFN therapy is an accepted alternative to hydroxyurea or busulfan in PV, and is particularly useful for younger patients or those who are pregnant (105, 106). In fact, IFN-α2 has been shown to induce haematological responses in about 80% of PV patients while also reducing splenomegaly, and relieving pruritus (107). A study of patients treated with IFN-α compared to a control group showed an MMR of 89%, with a stable circulating *JAK2* mutated allele (%V617F) in the other 11% of patients (108).

Recent trial data have demonstrated that 76% of advanced PV patients treated with IFN-α2a showed complete haematologic response, while the complete molecular response rate was 19% at 42 months (109). Similarly, good efficacy for pegylated IFN-α in hydroxyurea resistant patients was seen, with an overall response rate of 60% at 12 months (110). Significantly this trial was based on relatively broad inclusion criteria e.g., advanced age, prolonged disease duration, and a high prevalence of splenomegaly. However, 14% of patients discontinued therapy due to adverse events. However, the PROUD-PV and Continuation-PV trials revealed a need for prolonged IFN-α therapy as a means of reaching both molecular response and complete haematological response. Whilst therapy with ropeginterferon-alfa-2b (pegylated form of IFN-α2b) increased the proportion of patients achieving complete haematological response and improved disease burden after 36 months compared to those patients treated with hydroxyurea (53% *versus* 38%), this was not the case after shorter time periods (46% *versus* 51% at 12 months). Thus, there is a need for sustained treatment with IFN-α to achieve best response). Importantly, the toxicity profile associated with prolonged ropeginterferon-alfa-2b therapy was not significantly worse than that of hydroxyurea (111).

Primary Myelofibrosis (PMF) can be classified into two stages: pre-fibrotic (pre-PMF) and fibrotic PMF (overt-PMF) diagnosed by bone marrow morphology, fibrosis grade and clinical features including leukoerythroblastosis, anaemia and splenomegaly (112). Aside from HCT, there are limited treatment options for very high- and high-risk patients and 10-year survival is estimated at <13%. The use of JAK2 inhibitors is considered mostly palliative, and the treatment of clinical features includes the use of androgens, hydroxyurea and ruxolitinib (113). However, IFNs may provide a means of increasing the survival of patients with PMF. Preliminary results of a Phase II study using a combination therapy between pegylated IFN-α and ruxolitinib on 50 MPN patients (32 PV and 18 PMF patients), most of whom were resistant and/or intolerant to IFN-α as a monotherapy, saw complete haematologic responses in 44% and 58% of PV and PMF patients respectively (114). Despite a 20% discontinuation rate for this trial, there is clear indication that the inflammatory mediated toxicity which often limits IFN-α was reduced by ruxolitinib, known for its potent anti-inflammatory properties. It is also reported that levels of the V617F allele declined significantly in both groups. This supports previous findings that monitoring the %V617F is a reliable marker for MPNs and the detection of MRD.

Essential thrombocythaemia (ET) is a clonal MPN characterized by excess production of platelets and mature hyperlobulated megakaryocytes, with the presence of mutations in *JAK2*, *CALR*, or *MPL* and the absence of *BCR-ABL* (3). Most ET patients have a normal life expectancy, and the goals of treatment are the prevention of thrombotic and bleeding complications while minimizing the risk of progression, the effective control of systemic symptoms, and the appropriate management of complications and risk situations (105). Treatment of ET typically involves combinations of aspirin, ticlopidine, hydroxyurea and anagrelide to reduce platelet number and manage clotting (115, 116). IFN therapy in ET is also effective for reducing platelet numbers while also reducing the risk of thrombotic complications (107, 117) but does not appear to restore polyclonal haematopoiesis (118). Studies that will directly compare pegylated IFN-α2a to hydroxyurea in ET and PV are currently ongoing (110). Initial reports show an overall response rate of 69% for pegylated IFN-α2a in ET patients at 12 months, with acceptable safety profile (major thrombotic events at 1 year was 1%, with no major bleeding events).

A recent study comprising patients with PV, PMF or ET indicated IFN-α has a stronger effect on HSCs than mature blood cells. Within this compartment, those with *JAK2* V617F-mutated HSCs responded better than those with other mutations, such as *CALR.* This is thought to be a result of the *JAK2* V617F increasing the sensitivity of HSCs to IFN signalling, through increased ISG activation (119). Whilst it has been widely understood that IFN-α can reduce the HSC compartment through cell cycle activation and subsequent HSC exhaustion, recent findings suggest this is only the case in *JAK2* V617F mutated HSCs. In contrast, JAK2 wild-type HSCs generally remain quiescent in the presence of IFN-α. This is consistent with the ability of IFN-α to arrest the cell cycle in bulk tumor cells (48). Current understanding suggests IFN-α-mediated promotion of *JAK2* V617F-mutated HSC cell cycle not only exhausts the cells, but uses induces differentiation to the erythroid lineage, making them susceptible to IFN-α-mediated apoptosis (120). Nevertheless, the effects of IFN-α demonstrated here may not be wholly specific to the V617F mutant, since stem cell derived erythrocytes containing *JAK2* with exon 12 mutations (also causing constitutive JAK2 activation, as seen in PV) were also sensitive to IFN-α treatment (121).

## Conclusions & Future Directions

Similar to symptoms experienced during normal IFN release in the presence of a foreign pathogen, acute toxicity to IFN therapy usually presents as flu-like symptoms including nausea, fatigue, and myalgia (122). Most side effects associated with IFN therapy are dose-dependent, and these can be mitigated by administering low dose pegylated-IFNs in addition to managing inflammation with NSAIDs. Acute toxicity is experienced by nearly all patients, while sub-acute and chronic side effects have been reported in almost all organ systems throughout the body. A 2018 study conducted by Mo et al. using IFN-α for the treatment for MRD in AML for example, reported that all patients reported transient fevers and six patients (14%) showed grade ≥3 toxicity, two of which were haematological (77). A study of CML patients treated with low-dose pegylated IFN-α reported that 20% and 25% of patients experienced grade 3-4 neutropenia and thrombocytopenia in the first 12 months respectively (96). While some toxicity is still observed, pegylated IFNs show increased patient tolerance, and modify the immunological, pharmacokinetic and pharmacodynamic properties of the drug to minimize off-target adverse effects and increase efficacy (123). Pegylated IFN-α lengthens plasma half-life and reduces sensitivity to proteolysis, allowing it to be administered less frequently (122).

It is important that other adverse events in IFN therapy including flu-like symptoms and myalgia are also controlled using anti-inflammatory drugs. A common trend throughout the studies discussed herein is a high percentage of patients who do not complete IFN therapy because it is not well tolerated alongside chemotherapy, which can be as high as 61% of patients discontinuing treatment (54).

Another confounding factor for IFN therapy is the variability of the bone marrow niche between patients. IFN therapy relies on the innate, non-specific immune system to induce an immune response meaning it is not well tailored to one specific individual. This may partially explain why the responsiveness to IFN therapy in a small patient group varies greatly. To account for this, larger studies such as the TIGER study (98) are vital to better understand the causes for intolerance and resistance to therapy.

There is also a considerable economic barrier for uptake of IFN therapy. Analysis has shown that imatinib is a cost-effective first line therapy *versus* IFN-α plus low-dose cytarabine for newly diagnosed CML (124). If significant cohorts of patients will ultimately discontinue IFN therapy in favour of TKIs, then coupled with cost-based concerns, then IFNs become less favourable drugs to develop.

**Figure 2:**
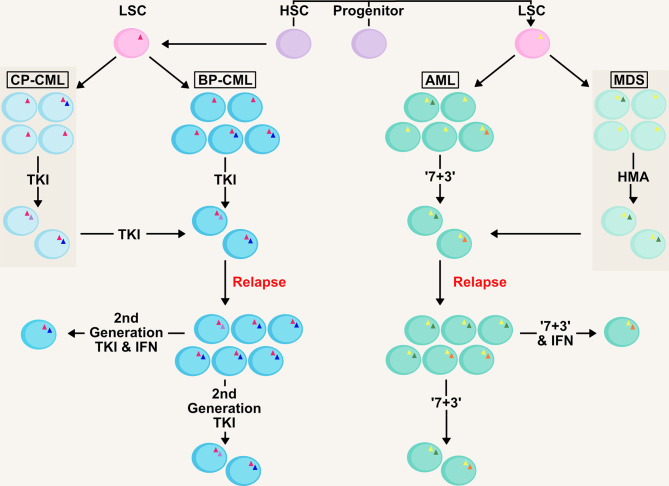
Management of Clonality and MRD in AML/CML with IFN therapy. AML and CML are clonal diseases, presenting at diagnosis with a major immature blast clone and minor clones (denoted by mutations as triangles within cells) which develop due to mutations in the haematopoietic stem and progenitor pool generating the LSC. Initial therapy in both chronic-phase (CP-CML) and blast-phase (BP-CML) patients with a first-generation tyrosine kinase inhibitor (TKI) against the *BCR-ABL* oncogene (pink triangle), such as imatinib, will reduce the bulk of CML blasts in the bone marrow and peripheral blood. However, it is not uncommon for resistant clones to persist that are refractory to this treatment. Later generation TKIs (e.g. nilotinib, dasatanib, ponatinib) may eradicate these clones but some mutations (e.g. *BCR-ABL* T315I; green circle) drive resistance to novel TKIs. Similarly, initial ‘7+3’ therapy in AML (7 day treatment with the pyrimidine analogue cytarabine, in conjunction with an anthracycline, such as daunorubicin, for the first 3 days) and HMA-therapy (hypomethylating agent) in MDS will eradicate the bulk of leukaemic blasts (such as those containing initiating mutations; yellow triangle) in the patient. However, clones refractory to these therapies (including those with signalling mutations; blue and orange circles) will drive relapse in MDS/AML patients, as well as transformation from MDS to AML. Management of measurable residual disease (MRD) in myeloid malignancies is critical to preventing relapse. Therapies based on IFNs could target the clones resistant to front-line therapies and deliver longer remissions.

It is apparent in AML that carefully targeted IFN therapy can provide a significant improvement to patient outcome especially in the management of MRD (27, 77, 78). The sensitization of leukaemic cells in the bone marrow niche by IFNs to classic chemotherapy agents will possibly improve the prospects of many AML patients should the treatment become more widely available (**Figure 2**), especially in patients post-HCT for the eradication of MRD. This is an extremely important goal, as multiple studies have shown the presence of MRD is associated with increased relapse risk and shorter survival in both paediatric and adult AML (71).

Similarly in CML, further development of IFN therapy provides an opportunity alongside current standards. Continuing focus on development of novel TKIs targeting BCR-ABL has meant that IFN therapy has not advanced greatly over the last decade for CML patients. There is potential for IFN therapy as a combination therapy with TKIs to sensitize cells and aid the action of the primary treatment. Just as in the case of AML, targeting the LSC to overcome resistance to TKIs is a fundamental goal and essential to achieving long-term remissions.

In PV and PMF, where the side effects of IFNs are tolerated, treatment using IFNs in combination with JAK2 inhibitors is certainly a possibility for the prevention of disease progression to other myeloid malignancies. As in AML, the use of IFNs for the treatment of PV and PMF to control MRD post-HCT would be beneficial for patients.

In conclusion, the benefits of using IFN therapy for the treatment of myeloid malignancies warrants further research. Despite intolerance seen in many small studies, IFNs are to be considered as an alternative therapy to traditional chemotherapy regimens or in combination with existing regimens for the sensitization of leukaemic cells to treatment and the reduction of MRD. It is important that other formulations of IFNs are developed in addition to pegylation, as this will determine whether pharmaceutical companies recognize this therapy as a reliable candidate for future treatment – the findings of large, ongoing Phase III trials will become a critical turning point. In short, the re-emergence of IFN therapy does indeed provide new hope for leukaemia patients, more specifically in the treatment of MRD.

## Author Contributions

JW conceived the study. FH, JJ, and JW wrote the manuscript. FH and JW produced the figures. YF and LD edited and contributed to the final submitted manuscript, and provided critical insights. All authors contributed to the article and approved the submitted version.

## Funding

JW and LD are supported by North West Cancer Research.

## Conflict of Interest

The authors declare that the research was conducted in the absence of any commercial or financial relationships that could be construed as a potential conflict of interest.

## Publisher’s Note

All claims expressed in this article are solely those of the authors and do not necessarily represent those of their affiliated organizations, or those of the publisher, the editors and the reviewers. Any product that may be evaluated in this article, or claim that may be made by its manufacturer, is not guaranteed or endorsed by the publisher.

## Abbreviations

ABL, Abelson proto-oncogene; AML, Acute Myeloid Leukaemia; ara-C, Cytarabine; bFGF, basic fibroblast growth factor; BCR, Breakpoint cluster region gene; cGAMP, 2’3’-Cyclic GMP-AMP; cGAS, cyclic GMP-AMP synthase; CML, Chronic Myeloid Leukaemia; cMyc, cellular Myc protein; COX, Cyclooxygenase; DNA, Deoxyribonucleic acid; DR5, Death receptor 5; ET, Essential thrombocythaemia; GVHD, Graft versus host disease; GVL, Graft versus leukaemia; HCT, Haematopoietic stem cell transplant; HSCs, Haematopoietic stem cells; IFNAR1/2, Interferon-alpha/beta receptor 1/2; IFNs, Interferons; IFN-α/β/γ, Interferon alpha/beta/gamma; IRF3/9, Interferon regulatory factor 3/9; ISGF3, Interferon-stimulated gene factor 3; ISGs, Interferon-stimulated genes; ISREs, Interferon-stimulated response elements; IκB, Inhibitor of kappa B kinase; JAK1/2, Janus Kinase 1/2; LSCs, Leukaemic stem cells; MDS, Myelodysplastic syndrome; MMR, Molecular response rate; MPNs, Myeloproliferative neoplasms; MRD, Measurable/minimal residual disease; MSCs, Mesenchymal stromal cells; NF-κB, Nuclear factor-kappa B; NSAIDs, Non-steroidal anti-inflammatory drugs; PAMPs, Pathogen-associated molecular patterns; PMF, Primary myelofibrosis; PV, Polycythaemia Vera; Rig-G, Retinoic acid-induced gene G; RUNX1, Runt-related transcription factor 1; STAT1/2, Signal transducer and activator of transcription 1/2; STING, Stimulator of interferon genes; TKIs, Tyrosine Kinase Inhibitors; TNF-α, Tumour necrosis factor alpha; TRAIL, Tumour necrosis factor-related apoptosis inducing ligand; TYK2, Tyrosine kinase 2; VEGF, vascular epidermal growth factor.
